# Stent-based electrode for radiofrequency ablation in the rat esophagus: a preliminary study

**DOI:** 10.1038/s41598-022-23472-7

**Published:** 2022-11-09

**Authors:** Dong-Sung Won, Yubeen Park, Jinsu An, Dae Sung Ryu, Jeon Min Kang, Ji Won Kim, Song Hee Kim, Chu Hui Zeng, Hongbae Kim, Hyung-Sik Kim, Jung-Hoon Park, Sang Soo Lee

**Affiliations:** 1 Biomedical Engineering Research Center, Asan Institute for Life Sciences, Asan Medical Center, 88 Olympic-Ro 43-Gil, Songpa-Gu, Seoul, 05505 Republic of Korea; 2 Department of Biomedical Engineering, School of ICT Convergence Engineering, College of Science & Technology, Konkuk University, Chungju, Republic of Korea; 3 Department of Biosystems & Biomaterials Science and Engineering, Seoul National University, Seoul, 08826 Republic of Korea; 4 Department of Mechatronics Engineering, School of ICT Convergence Engineering, College of Science & Technology, Konkuk University, Chungju, Republic of Korea; 5 Department of Gastroenterology, Asan Medical Center, University of Ulsan College of Medicine, 88 Olympic-Ro 43-Gil, Songpa-Gu, Seoul, 05505 Republic of Korea

**Keywords:** Gastrointestinal diseases, Biomedical engineering, Preclinical research

## Abstract

Endoluminal radiofrequency (RF) ablation has been widely used as a safe and effective treatment for Barrett’s esophagus. However, inadequate RF ablation may occur due to insufficient contact between the electrode and target tissues. Herein, a stent-based monopolar RF electrode (SE) was developed to evenly deliver RF energy to the inner wall of the rat esophagus. The optimal RF parameters were evaluated in the exposed rat esophagus. The temperature in the rat esophagus reached 70 ℃ in 89 s at 30 W, 59 s at 40 W, and 34 s at 50 W. The technical feasibility and efficacy of RF ablation using SE were evaluated based on changes in histological transformation and immunohistochemical parameters of tissues compared at immediately, 1 and 2 weeks after the procedure. The degrees of inflammatory cell infiltration, fibrotic changes, TUNEL, and HSP70 in the RF-ablated rat esophagus were significantly higher than compared with sham control (all *p* < 0.05). TUNEL-positive deposition gradually decreased, but HSP 70-positive deposition maintained a similar level for 2 weeks. The stent-based RF ablation was technically feasible and effective in evenly inducing thermal damages to the rat esophagus. The RF ablation system using the SE may represent a promising treatment for endoluminal malignancies.

## Introduction

Radiofrequency (RF) ablation has been widely used as a safe and effective modality for the treatment of various malignancies^1,2^. Endoluminal RF electrodes such as catheter- or balloon-based have been developed for the management of inoperable biliary and pancreatic ductal cancers presenting with obstructive disorders, as well as for Barrett’s esophagus^3–7^. A catheter-based electrode has the advantage of being easy to handle and RF ablation catheter in malignant biliary obstructions has provided beneficial effects on survival and stent patency in clinical trials^8,9^. However, these electrodes can neither fully contact the target tissue nor be adjusted for luminal organs of different diameters^10,11^. Balloon-based electrodes can be applied to various forms of stricture through inflation and adjustment of the diameter of the balloon. Conversely, however, if the balloon size is large, the wall of the esophagus may stretch and cause balloon migration. Moreover, if complete ablation is not achieved after the first procedure, repeated procedures may be required to perform circumferential RF ablation^5,6^.

RF ablation induces thermal injury to the tissue through electromagnetic energy deposition. Whilst the contiguous tissues on the electrode undergo the highest current and heat shock due to reduced electrical conductivity of the tissues^12,13^, the tissues away from the electrodes are heated mainly by thermal conduction. Therefore, inadequate RF ablation may occur due to insufficient contact between the electrode and the target tissues especially when the strictures are soft and non-tight, and the target tissues have an irregular shape^14–16^. Recently, a stent-based monopolar RF electrode (SE) was developed to increase the contact area with the endoluminal tissues to enhance the therapeutic effects by an even RF ablation. Therefore, the purpose of this study was to investigate the technical feasibility and efficacy of RF ablation with use of a newly developed SE in the rat esophagus.

## Methods

### Preparation of the stent-based RF electrode

**Figure 1:**
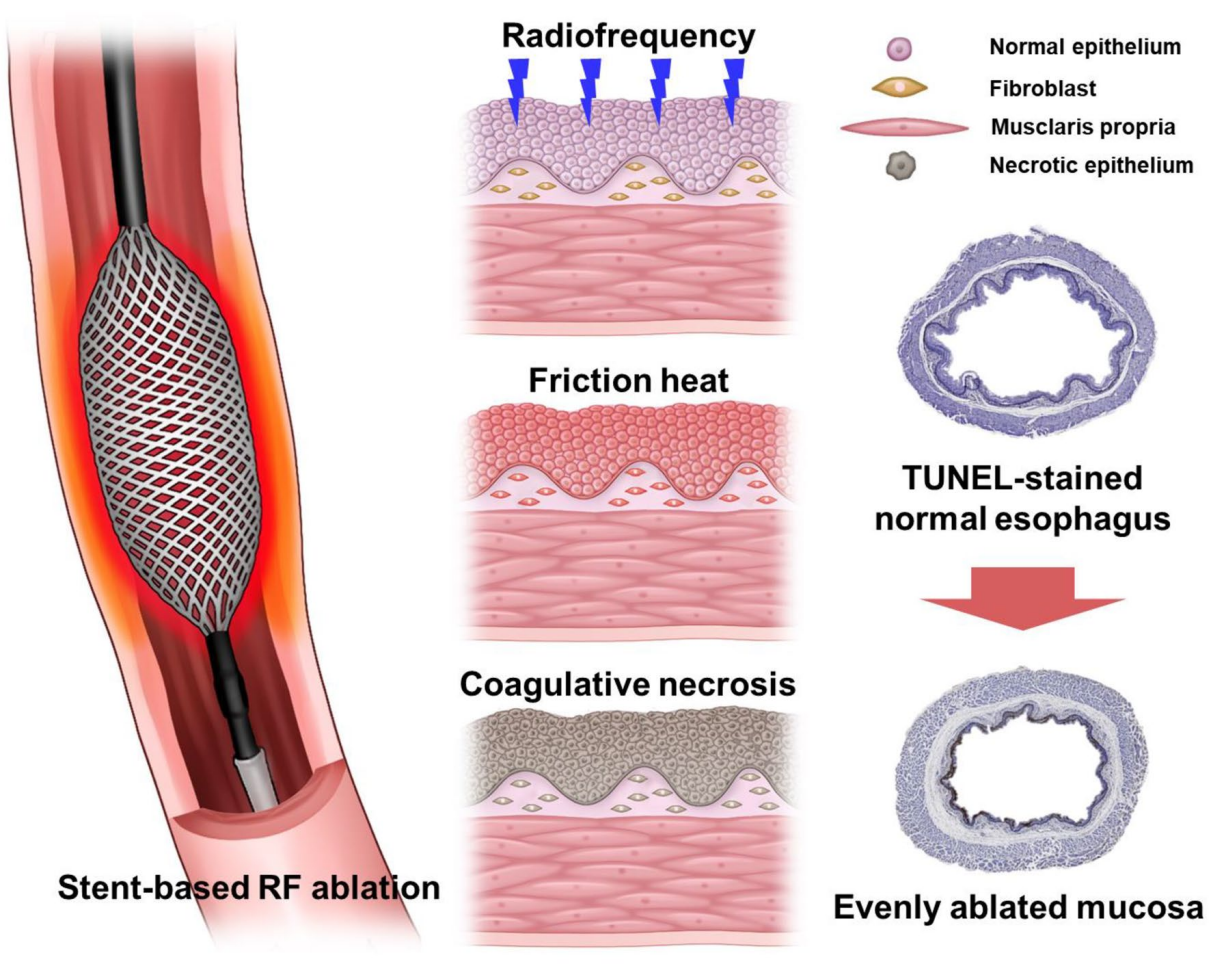
Schematic illustration of the radiofrequency ablation using the stent-based electrode in the rat esophagus and representative TUNEL-stained images indicating the resulting therapeutic effects.

The SE was designed to evenly deliver RF energy to the inner wall of the rat esophagus (S&G biotech, Ltd., Yongin, Korea) (Fig. 1). The SE was braided using 32 nitinol wires with a thickness of 0.09 mm. The diameter of the SE was 5 mm, and the length was 3 mm. The delivery system was a 750-mm-long 8-Fr catheter and comprised an insulating outer sheath and a pusher with a guiding olive tip. The ends of the SE were fixed in the delivery system for removal immediately after the RF ablation procedure (Supplementary Fig. 1). The SE was connected to a RF ablation system (CoATherm AKF200; APRO KOREA, Gunpo, Korea).

### Stent-based RF ablation-induced apoptotic effects in gastric cancer cells

The gastric cancer cell line (SNU-NCC-24; Korean Cell Line Bank, Seoul, Korea) was used to validate the apoptotic effects of stent-based RF ablation. The cells were incubated in RPMI-1640 (Welgene Inc, Gyeongsan, Korea) containing 5% fetal bovine serum and supplemented with 1% antibiotics (penicillin [100 U/ml]-streptomycin [100 ug/ml]; Gibco, Life Technologies, Carlsbad, CA, US) in an incubator (KE72 ThermoForma 3111 CO_2_ Incubator; Thermofisher, Waltham, MA, US) at 37 °C and 5% CO_2_. The 1.0 × 10^5^ cells were aliquoted into microcentrifuge tube (AX-MCT-150-A; Corning®, NY, US) in 1.5 mL of culture media. The SE was dipped into these cell suspensions in the tubes and RF ablation was conducted at 480 kHz and 30, 40 and 50 Watts (W) for 30 s. The ablated cells were transferred to a 24-well plate (SPL life sciences Co., Ltd, Pocheon, Korea). After incubating for 6 h, the adherent cells were harvested. Aliquots of 100 µL of the collected cells were added to new microcentrifuge tubes supplemented with 100 µL of Muse Annexin V & Dead Cell Reagent (Luminex Corporation, Austin, TX, US), and incubated for 20 min at room temperature. Apoptotic cells were assayed with a Muse cell analyzer (Luminex Corporation, Austin, TX, US). The percentages of total apoptotic cells were calculated as the sum of early and late apoptotic cells. The temperature at the center of SE was measured by a thermal camera (FLIR A400; Teledyne FLIR, Wilsonville, OR, US).

### Animal study design

A total of 24 Sprague–Dawley rats weighing 250–263 g (mean weight, 258.5 g) were used in this study. Three rats were used to determine the optimal RF parameters, and the remaining 21 rats were used to investigate the efficacy of stent-based RF ablation in-vivo experiment.

Eighteen of the 21 rats received stent-based RF ablation to investigate the changes in the ablated esophageal tissue over time. The remaining three rats received a sham procedure. The eighteen rats were randomly sacrificed immediately, 1 week, and 2 weeks after the RF procedure (n = 6 in each group) by administering inhalable pure carbon dioxide. All rats were housed under the same environmental conditions with free access to food and water and a temperature of 24 ± 2 °C with a 12-h day-night cycle. Body weight was measured twice weekly until sacrifice.

### RF parameters of stent-based RF ablation in the rat esophagus

Stent-based RF ablation was performed in the surgically exposed rat esophagus to determine the optimal RF parameters for the rat esophagus. The SE was inserted through the mouth into the cervical esophagus of rats. Stent-based RF ablation was conducted at 30, 40, and 50 W to measure the time needed to reach 70 ℃, and the corresponding time was then set to ensure 70 ℃ was reached in-vivo. The impedance was recorded at 1 s interval during the delivery of the RF energy. The temperature was recorded at 1 s interval until the temperature had cooled to 40 ℃. All rats were immediately sacrificed by administering inhalable pure carbon dioxide for gross examination to confirm esophageal injuries.

### Stent-based RF ablation in the rat esophagus

The technical feasibility of stent-based RF ablation was evaluated in the rat esophagus. All rats were anesthetized by intramuscularly injecting 50 mg/kg zolazepam and 50 mg/kg tiletamine (Zoletil 50; Virbac, Carros, France) and 10 mg/kg xylazine (Rompun; Bayer HealthCare, Leverkusen, Germany). A 0.014-inch micro guidewire (Transcend; Boston Scientific, Watertown, MA, US) was then inserted through the mouth into the stomach under fluoroscopy guidance (MeteoR; NanoFocusRay Co., Iksan, Korea). An 8-Fr delivery system was advanced and the SE was fully expanded. RF energy was applied for 60 s at a power of 40 W and 480 kHz with 70 ℃. During the RF ablation, impedance changes of the rat esophagus was recorded at 1 s intervals. Antibiotics (gentamicin, 80 mg/2 mL; Shin Poong Pharm Ltd., Seoul, Korea) and analgesics (keromin, 30 mg; Ketorolac; Hana Pharm Ltd., Seoul, Korea) were routinely used for three days after the procedure.

### Histological examination

Histological examination was performed to observe morphological mucosal changes and to evaluate the degrees of inflammation and fibrotic changes. Extracted esophagus samples were fixed in 10% neutral buffered formalin for 24 h, embedded in paraffin, and transversely sectioned. The samples were stained with hematoxylin & eosin (H&E) and Masson’s trichrome (MT). H&E-stained sections were used to assess the thicknesses of the submucosal fibrosis, and the thickness of the epithelial layer were measured microscopically and averaged at eight points around the circumference. Inflammatory cell infiltration was subjectively determined in accordance with the distribution and density of the inflammatory cells (graded as 1, mild; 2, mild to moderate; 3, moderate; 4, moderate to severe; and 5, severe). The degree of collagen deposition was subjectively determined using MT-stained sections where 1 indicated mild, 2 mild to moderate, 3 moderate, 4 moderate to severe, and 5 severe.

### Immunohistochemistry

Immunohistochemistry (IHC) was performed to evaluate the extent of cell death and the presence of heat shock by RF ablation. IHC was conducted on paraffin-embedded tissue sections using terminal deoxynucleotidyl transferase mediated dUTP nick and labeling (TUNEL, ApopTag Peroxidase In Situ Detection kit; Millipore Co., Burlington, MA, US) and heat shock protein 70 (HSP70, LS-B3700-50; LifeSpan BioSciences Inc., Seattle, WA, US) primary antibodies. The extents of TUNEL and HSP70-positive deposition were subjectively determined (1, mild; 2, mild to moderate; 3, moderate; 4, moderate to severe; and 5, severe). All histologic analyses were performed with a digital slide scanner (Panoramic 250 FLASH III; 3D Histech Ltd., Budapest, Hungary) and measurements were obtained with a digital microscope viewer (CaseViewer; 3D Histech). Histological analysis was conducted by the consensus of three observers who were blind to the animal groupings.

### Statistical analysis

Statistical analysis was performed to detect the differences among the study groups. Data were expressed as a mean ± standard deviation (SD). Differences between the groups were analyzed using the Mann–Whitney *U* test and two-sample *t* test, as appropriate. *P* < 0.05 was considered statistically significant. All statistical analyses were performed using SPSS software, version 27.0 (SPSS, IBM, Chicago, IL).

### Ethics declarations

All experiments were performed in accordance with relevant named guidelines and regulations.

### Approval for animal experiments

The use of animals in this study was approved by the Institutional Animal Care and Use Committee of the Asan institute for Life Sciences (Seoul, Korea) (IACUC No. 2020-12-316) and conformed to US National Institutes of Health guidelines for the humane handling of laboratory animals. The study was carried out in compliance with the ARRIVE guideline.

## Results

### Stent-based RF ablation-induced apoptotic effects

**Figure 2:**
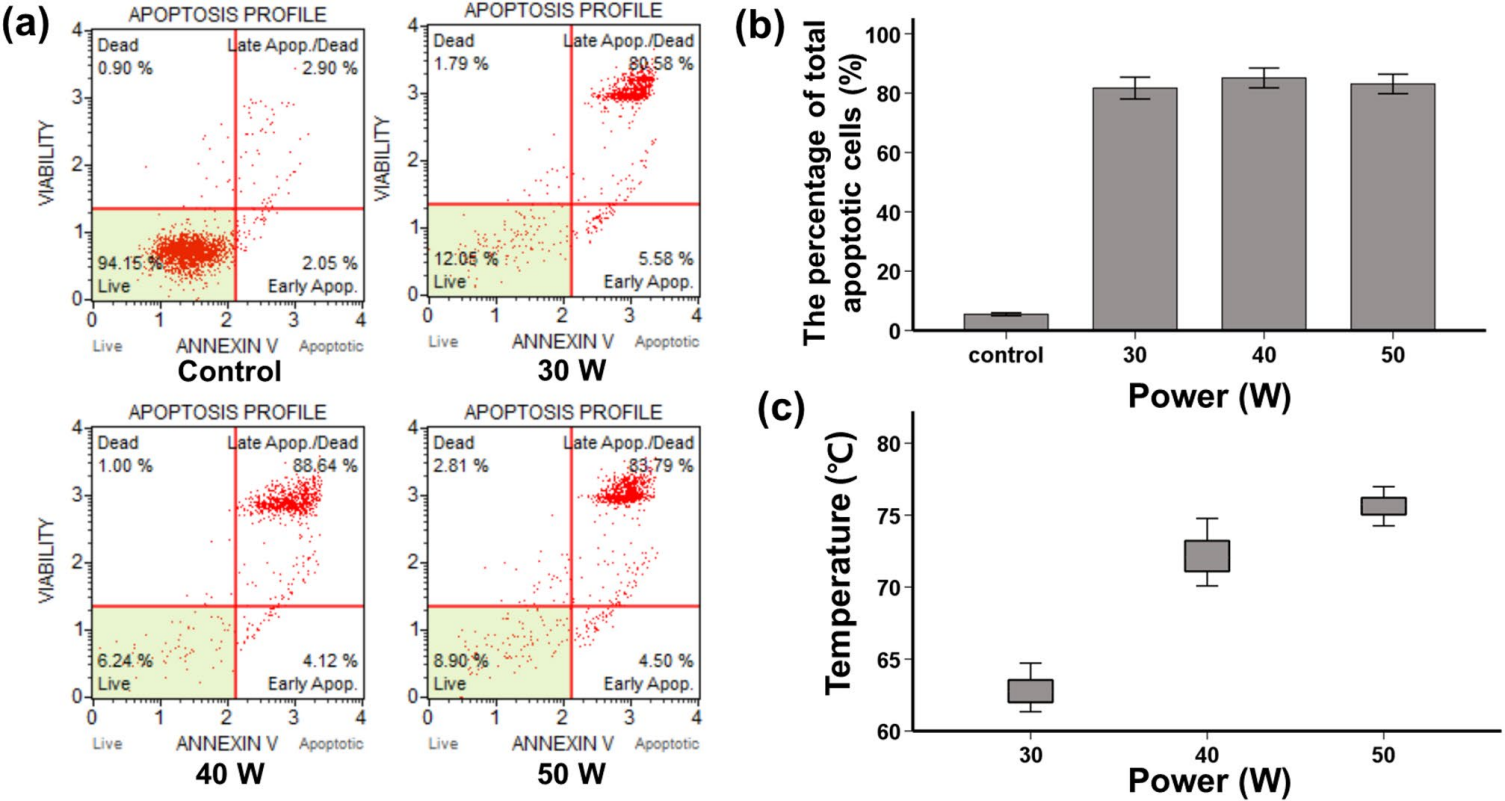
Radiofrequency (RF) ablation using a stent-based electrode in gastric cancer cells to verify apoptotic effects. (**a**) Reduction in the viable cell number was determined using an annexin V assay. (**b**) Graph showing the total quantity of apoptotic cancer cells (early + late apoptotic cells). (**c**) Maximum temperature in accordance with the power setting used for the RF ablation.

RF energy was successfully delivered to the gastric cancer cells through SE. The mean (± standard deviation, SD) maximum temperatures at 30, 40, and 50 W were 62.85 ± 1.09 ℃, 72.21 ± 1.50 ℃, 75.65 ± 0.86 ℃ respectively. There were no significant differences in the percentage of total apoptotic cells at 30 (81.71 ± 3.5%), 40 (85.13 ± 3.17%), or 50 W (83.10 ± 3.12%) (30 vs. 40 W, *p* = 0.107; 30 vs. 50 W, *p* = 0.483; 40 vs. 50 W, *p* = 0.292) (Fig. 2).

### Determination of stent-based RF parameter in the rat esophagus

**Figure 3:**
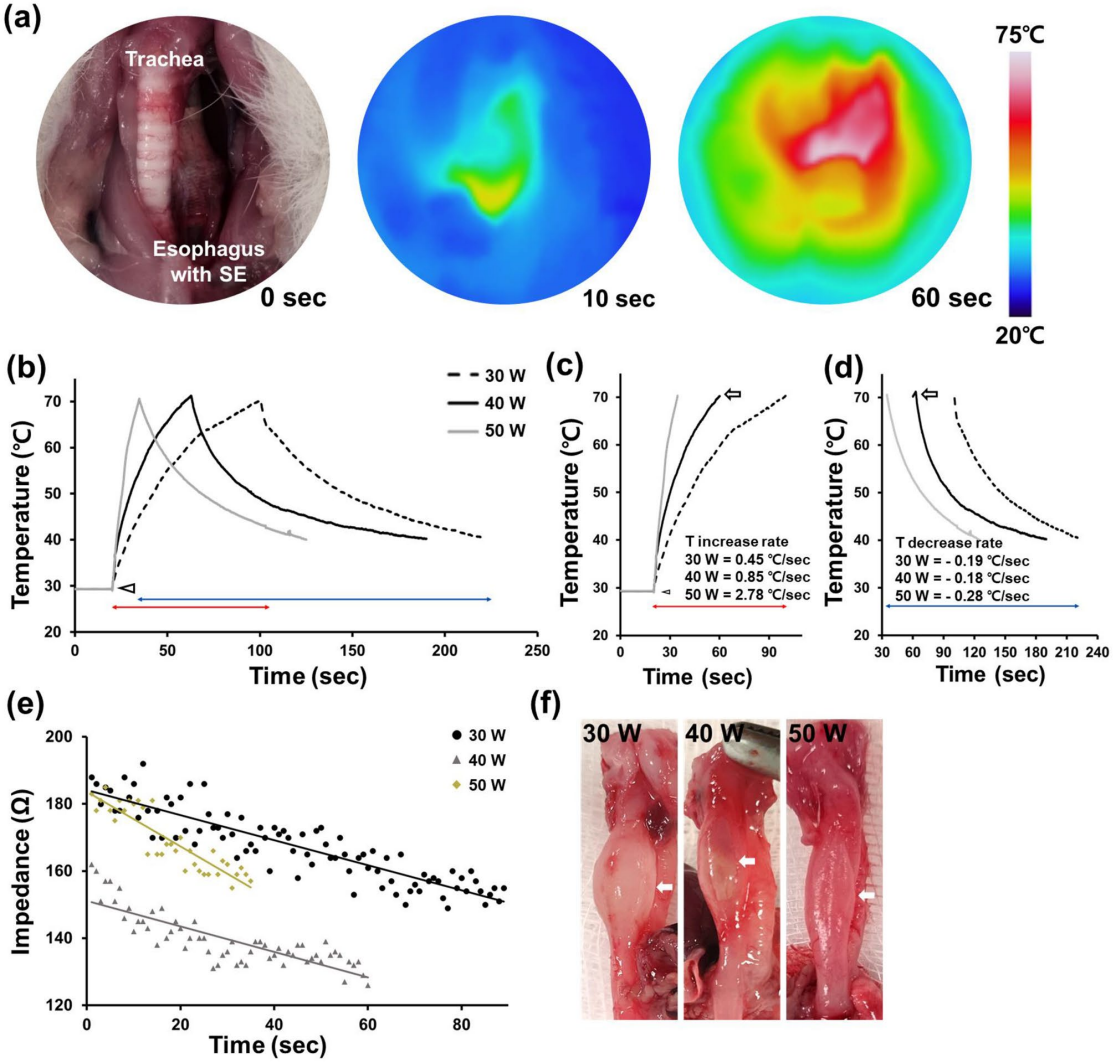
Temperature changes during radiofrequency (RF) ablation via the stent-based monopolar RF electrode (SE) in the rat esophagus. (**a**) Representative photographs and thermal images obtained during RF ablation of the rat esophagus through the SE at a 40 W power setting for 60 s. (**b**) Graph showing the recorded temperature changes during RF ablation at 30, 40 and 50 W. All RF energies were applied starting at the same time point (*arrowhead*) until the temperature reached 70 ℃ (*arrow*). (**c**) Initial heating temperature (*red line*). (**d**) Cooling temperature changes after the cessation of RF energy transfer (*blue line*). **(e)** Impedance profile changes during RF ablation in the exposed rat esophagus in accordance with different power settings at 30, 40, and 50 W. (**f**) Gross finding by various RF parameter in the extracted rat esophagus.

Stent-based RF ablation was successful in the exposed rat esophagus without any roll-off or esophageal perforation (Fig. 3). Mucosal injuries with a thinner esophageal wall were observed at 30, 40 and 50 W. The temperature in the rat esophagus reached at 70 °C in 89 s at 30 W, 59 s at 40 W, and 34 s at 50 W. The temperature gradually cooled to 40 °C after the cessation of RF energy in 212 s at 30 W, 189 s at 40 W, and 125 s at 50 W (Fig. 3b–d). The impedance level was decreased from 192 to 149 Ω at 30 W, 162 to 126 Ω at 40 W and 185 to 155 Ω at 50 W (Fig. 3e).

### Procedural outcomes of stent-based RF ablation in the rat esophagus

**Figure 4:**
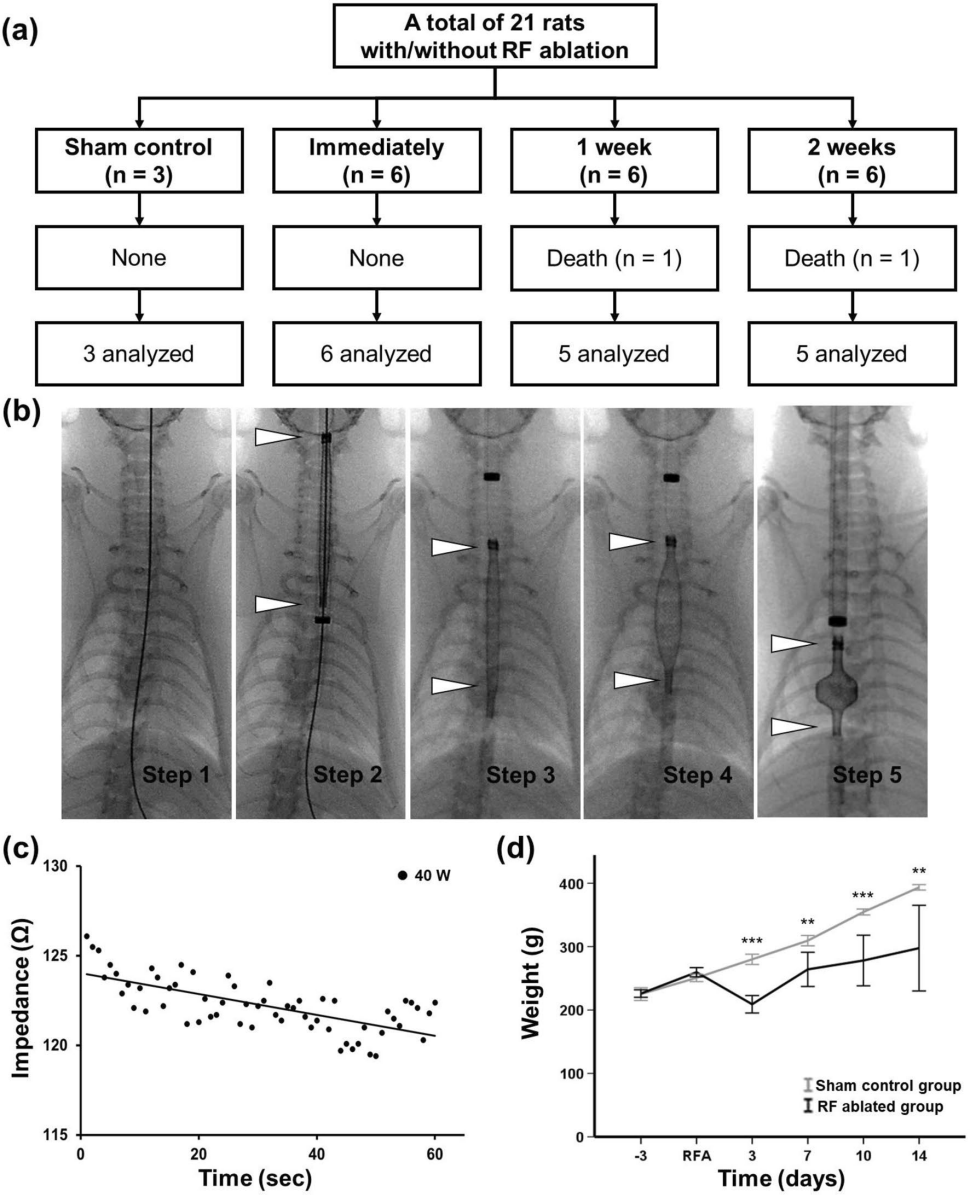
In-vivo experimental design, impedance, and weight changes. (**a**) Flow diagram of the in-vivo study design and follow-up. (**b**) Radiographic images showing the technical steps for the radiofrequency (RF) ablation using a stent-based monopolar RF electrode (SE) in the rat esophagus. Step 1, a micro guidewire was inserted through the esophagus into the stomach; step 2, a SE loaded delivery system (*arrowheads*) was advanced over the guidewire; step 3, the olive tip of the SE (*arrowheads*) was placed at the margin of the diaphragm under fluoroscopic guidance; step 4, the adjustable SE (*arrowheads*) was deployed with a diameter of 4 mm and a length of 3 mm to fully attach the electrode to the entire inner wall of the rat esophagus. (**c**) Impedance changes during RF ablation at 40 W for 60 s in the rat esophagus. (**d**) The body weight changes after RF ablation. *p < 0.05, **p < 0.01, ***p < 0.001.

Stent-based RF ablation under fluoroscopic guidance was technically successful in 16 (88.8%) of the 18 rats. Two rats died of dyspnea because the 8-Fr SE delivery system pressed the trachea during the procedure and were excluded from this study. The remaining 16 rats survived until the end of the study (Fig. 4). The mean impedance gradually decreased from 126.1 ± 26.7 Ω to 119.8 ± 18.8 Ω during RF ablation in the rat esophagus (Fig. 4c). The body weights of the treated rats significantly decreased at 3 days after RF ablation (209.00 ± 32.51 g) compared with the sham control (276.33 ± 32.51 g, *p* < 0.001), and gradually recovered in the ablated animals (Fig. 4d).

### Histological findings

**Figure 5:**
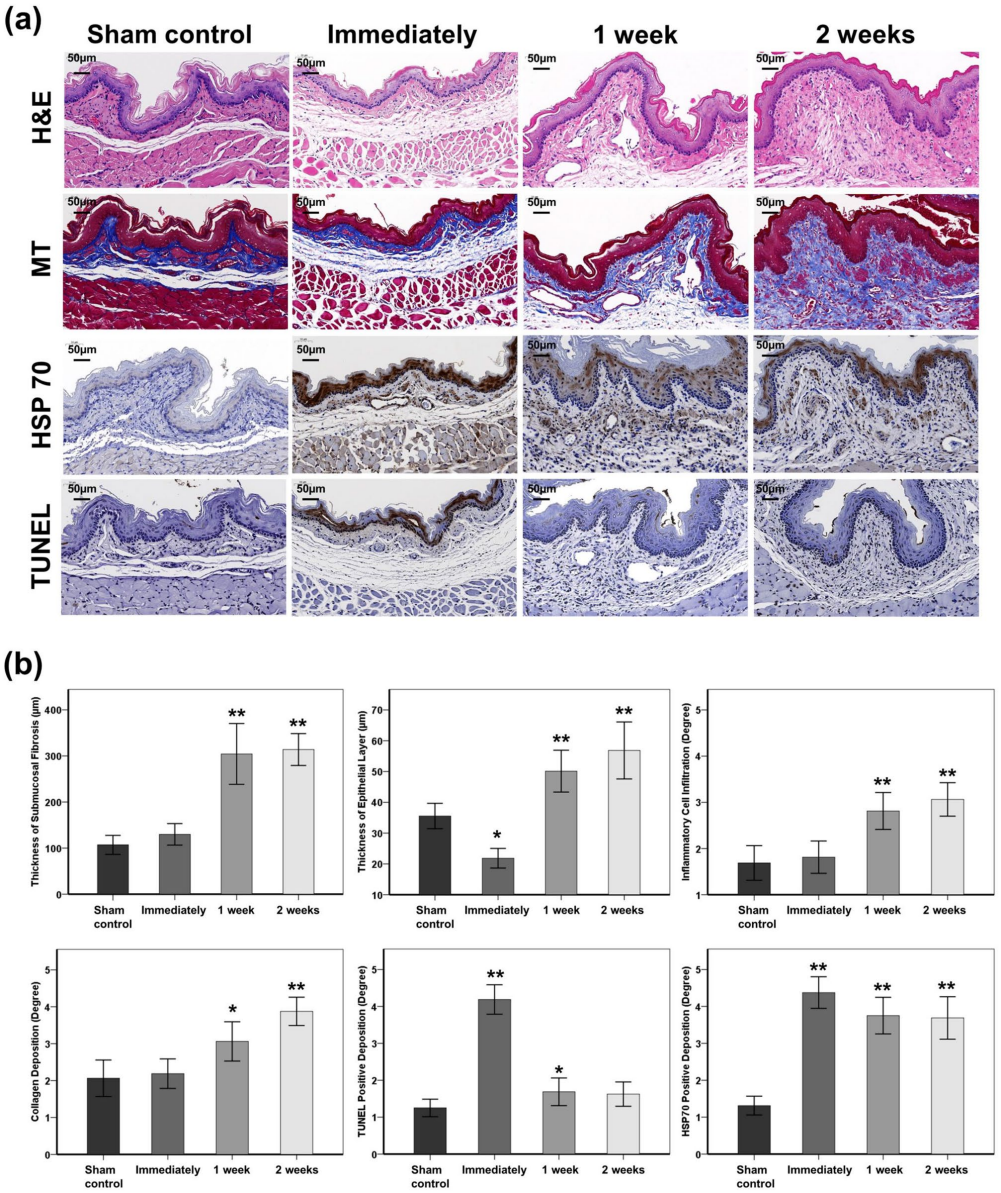
Representative microscopic images and serial histological findings at the indicated time points after radiofrequency (RF) ablation via the stent-based monopolar RF electrode (SE). (**a**)Representative microscopic images of hematoxylin & eosin-, Masson’s trichrome-, heat shock protein 70 (HSP70)-, and terminal deoxynucleotidyl transferase mediated dUTP nick and labeling (TUNEL)-stained tissue slides. (**b**) Graphs of the histologic findings indicating serial changes to the thicknesses of the submucosal fibrosis and epithelial layers, the degrees of inflammatory cell infiltration, collagen deposition levels, and TUNEL and HSP 70 positivity. *p < 0.05, **p < 0.001.

The mean (± SD) thickness of the submucosal fibrosis slightly increased immediately after RF ablation compared to the sham control without statistical significance (129.92 ± 43.84 μm vs. 107.04 ± 38.71 μm, *p* = 0.128). This change was significantly increased at 1 week (304.43 ± 123.94 μm, *p* < 0.001) and 2 weeks (313.94 ± 64.83 μm, *p* < 0.001) after RF ablation compared with the sham control. The mean thickness of the epithelial layer (21.84 ± 5.97 μm) was significantly decreased immediately after RF ablation compared with the sham control (35.54 ± 7.73 μm, *p* < 0.001). The thickness of the epithelial layer significantly increased at 1 week (50.12 ± 12.75 μm, *p* < 0.001) and 2 weeks (56.85 ± 17.35 μm, *p* < 0.001) after RF ablation compared with the sham control. The inflammatory cell infiltration levels gradually increased over time after RF ablation at immediately (1.81 ± 0.66, *p* = 0.607), 1 week (2.81 ± 0.75, *p* < 0.001), and 2 weeks (3.06 ± 0.68, *p* < 0.001) after the procedure compared with the sham control (1.69 ± 0.70). The degree of collagen deposition also gradually increased over time at immediately (2.19 ± 0.75, *p* = 0.678), 1 week (3.06 ± 0.99, *p* = 0.006), and 2 weeks (3.87 ± 0.72, *p* < 0.001) compared with the sham control (2.06 ± 0.93). The TUNEL-positive depositions significantly increased immediately after RF ablation compared with the sham control (4.19 ± 0.75 vs. 1.25 ± 0.45, *p* < 0.001) and gradually decreased at 1 week (1.69 ± 0.70, *p* = 0.044) and 2 weeks (1.63 ± 0.62, *p* = 0.059) after the procedure. The mean HSP70-positive deposition levels also significantly increased immediately after RF ablation compared with the sham control (4.38 ± 0.81 vs. 1.31 ± 0.48, *p* < 0.001). This difference was maintained at 1 week (3.75 ± 0.93, *p* < 0.001) and 2 weeks (3.69 ± 1.08, *p* < 0.001) after the procedure compared with the sham control (Fig. 5 and Supplementary Fig. 2).

## Discussion

The SEs were successfully prepared by the braiding technique using multiple nitinol wires and the recapturable delivery system was 8-Fr in diameter. Although two of the 18 rats died of dyspnea caused by compression of the upper respiratory tract due to thick delivery system in Sprague–Dawley rats mean weighting 258.5 g, RF ablation using SE was successfully performed, and evenly ablated esophageal mucosa was observed in the remaining rats. The histological results in our study demonstrated that circumferential heat-induced fibrotic changes were observed with increased collagen deposition and enhanced thickness of the epithelial layer with submucosal fibrosis. Consistently, IHC findings in the RF-ablated rat esophagus revealed that cellular apoptosis along with HSP70 expression significantly increased compared with the sham control group. These findings support the SE-based RF ablation was technically feasible and effective in the rat esophagus.

Our present results revealed that the stent-based RF ablation successfully induced thermal damages to the rat esophagus leading to cellular apoptosis and heat-mediated tissue injuries. The SE enabled fully contact with the rat esophageal wall, thereby allowing uniform RF energy delivery to the target lesion. A nitinol stent-based RF electrode has several advantages over other electrodes that are currently in use for endoluminal RF ablation. First, because it has a cylindrical structure in the form of a stent, it is suitable for endoluminal organs such as the esophagus, duodenum, bile ducts, and blood vessels. Second, the nitinol wires confer self-expanding properties and high flexibility which may be very useful to treat curved lesions^17,18^. Of note, our findings suggest that SE made of nitinol wires may be effective for circumferential ablation of the non-vascular luminal organs with significantly better therapeutic effects^19–21^, and whether it performs well in highly tortuous structures is worth further investigation.

The annexin V assay was used in this study to evaluate whether the reduction in viable cell number following stent-based RF ablation in gastric cancer cell lines. Our RF ablation system has been shown to have induce effects in human cancer cells. Although there were no significant differences at different power settings in the current findings, the feasibility of the stent-based RF ablation was confirmed considering that apoptosis of tumor cells has been shown to occur above 60 °C via local thermal effects^22–24^. Proper temperature measurement in the cellular structure and tissue means critical to achieving the desired clinical effects from RF energies^24^. Given that a temperature of 70℃ is usually set for the gastrointestinal tract in the clinic, this temperature was maintained in this present study^2,25^. However, since a thermal feedback system can hardly be installed in the SE, the temperature in the RF-ablated esophagus was continuously recorded using a thermal camera instead. Moreover, as other studies have reported that the tissue temperature rises, ion mobility within the heated tissue increases, causing a higher current flow and a lower impedance, it could be a specific marker of local tissue heating at a given target site during the RF ablation procedure^26–28^. Therefore, it could be expected that a certain ablation region would be achieved despite the absence of a thermal feedback system, since the difference in impedance during the RF ablation time was not large as 10–30 Ω in our current study^29–31^. The optimization of the RF energy and ablation time may affect efficient RF ablation.

There were several limitations to our study of note. First, the sample size in the study groups was small and it may decrease the statistical power of the study. Second, the absence of a temperature feedback system which directly applied to the SE was the major obstacle to perform efficient RF ablation. It is necessary to confirm the thermal feedback to the generator for an accurate evaluation of RF ablation in the future. Third, RF ablation using the SE was conducted in the normal rat esophagus. Evaluation with esophageal cancer rat model would be necessary in the future. Fourth, only a few markers were used for the histological examinations.

In conclusion, although additional studies are needed to further validate the current data, this study successfully demonstrated stent-based RF ablation was technically feasible and effective to evenly induce thermal damages to the rat esophagus. The SE can maximize the RF ablation-induced therapeutic effects by fully contacting the inner wall of the rat esophagus. The stent-based RF ablation system may represent a promising new approach for the treatment of endoluminal malignancies in non-vascular luminal organs.

## Supplementary Information

Supplementary Information.

## Data Availability

Due to its proprietary nature, neither the data nor the source of the data can be made available publicly, but the data presented in this study are available on request from the corresponding author.
