# Supplementary material for: Stent-based electrode for radiofrequency ablation in the rat esophagus: a preliminary study
Source: Sci Rep. 2022 Nov 9;12:19135. doi: 10.1038/s41598-022-23472-7 (PMC9646798; doi:10.1038/s41598-022-23472-7)
Supplement: Supplementary file 1 — Supplementary Information. [file 41598_2022_23472_MOESM1_ESM.docx]

SUPPLEMENTARY MATERIALS

**Novel Stent-based Electrode for Radiofrequency Ablation in the Rat Esophagus: A Preliminary Study**

*Dong-Sung Won^1†^, Yubeen Park^1,2†^, Jinsu An^3^, Dae Sung Ryu^1^, Jeon Min Kang^1^, Ji Won Kim^1,2^, Song Hee Kim^1,2^, Chu Hui Zeng^1^, Hongbae Kim^4^, Hyung-Sik Kim^5^,* *Jung-Hoon Park^1*^, Sang Soo Lee^2*^*

*^1^Biomedical Engineering Research Center, Asan Institute for Life Sciences, Asan Medical Center, 88 Olympic-ro 43-gil, Songpa-gu, Seoul, 05505, Republic of Korea*

*^2^Department of Gastroenterology, Asan Medical Center, University of Ulsan College of Medicine, 88 Olympic-ro 43-gil, Songpa-gu, Seoul, 05505, Republic of Korea*

*^3^Department of Biomedical Engineering, BK21 Plus Research Institute of Biomedical Engineering, School of ICT Convergence Engineering, College of Science and Technology, Konkuk University, Chungju, 27478, Republic of Korea*

*^4^Department of Biosystems & Biomaterials Science and Engineering, Seoul National University, Seoul, 08826, Republic of Korea*

*^5^Department of Biomedical Mechatronics Engineering, School of ICT Convergence Engineering, College of Science & Technology, Konkuk University, Chungju, 27478, Republic of Korea*

^†^D.-S.W. and Y.P. contributed equally to this work and are co-first authors.

^*^J.-H.P. and S.S.L. contributed equally to this work and are the co-corresponding authors.

**Correspondence:**

Jung-Hoon Park, Ph.D.^1^ and Sang Soo Lee, M.D. Ph.D.^2^

^1^Biomedical Engineering Research Center, Asan Institute for Life Sciences, Asan Medical Center, 88 Olympic-ro 43-gil, Songpa-gu, Seoul, 05505, Republic of Korea

Tel: 82-2-3010-4123 Fax: 82-2-476-0090

E-mail: [jhparkz@amc.seoul.kr](mailto:jhparkz@amc.seoul.kr)

^2^Department of Gastroenterology, Asan Medical Center, University of Ulsan College of Medicine, 88 Olympic-ro 43-gil, Songpa-gu, Seoul 05505, Republic of Korea

Tel: 82-2-3010-3187 Fax: 82-2-476-0090

E-mail: [ssleedr@amc.seoul.kr](mailto:ssleedr@amc.seoul.kr)


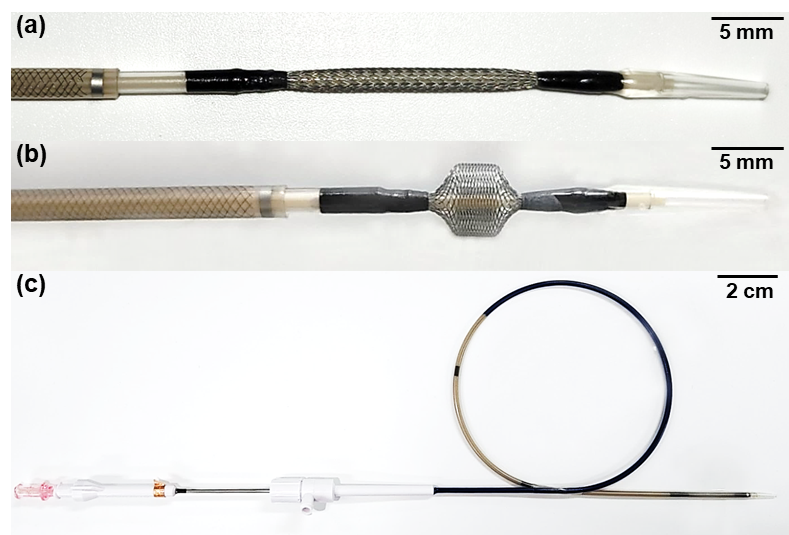


**Supplementary Figure 1.** The stent-based electrode (SE) and delivery system for RF ablation. Photograph showing **(a)** the SE was crimped before expansion and **(b) t**he SE was fully expanded to delivery uniform RF energy. **(c)** The delivery system was developed for removal immediately after RF ablation.


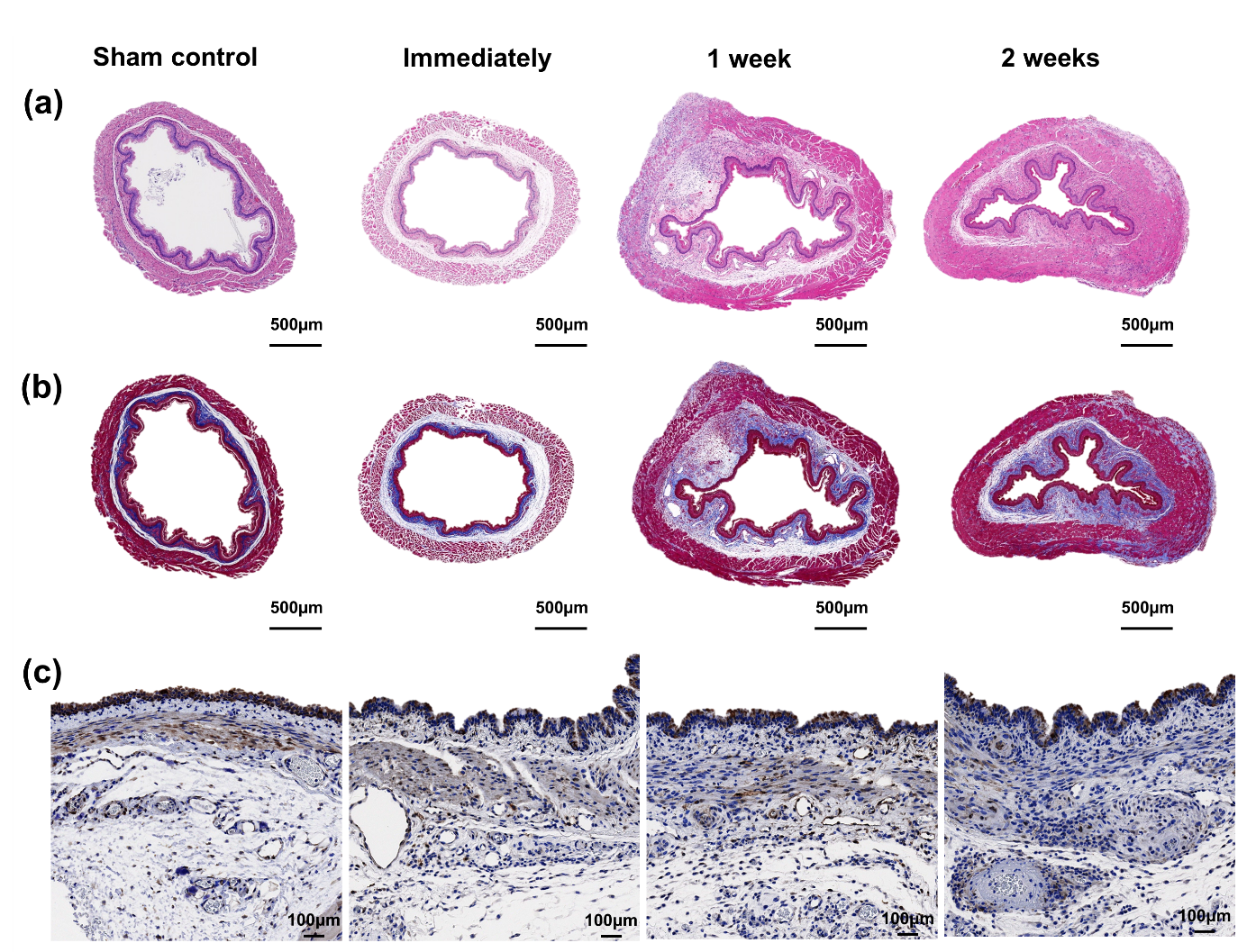


**Supplementary Figure 2.** Histological changes in the entire rate after RF ablation via the SE. Representative **(a)** H&E and **(b)** MT-stained microscopic images of histological sections from the indicated animal study groups in esophagus after RF ablation.
